# Loss of arylformamidase with reduced thymidine kinase expression leads to impaired glucose tolerance

**DOI:** 10.1242/bio.013342

**Published:** 2015-10-02

**Authors:** Alison J. Hugill, Michelle E. Stewart, Marianne A. Yon, Fay Probert, I. Jane Cox, Tertius A. Hough, Cheryl L. Scudamore, Liz Bentley, Gary Wall, Sara E. Wells, Roger D. Cox

**Affiliations:** 1 Mammalian Genetics Unit, Medical Research Council Harwell, Oxford, Oxfordshire OX11 0RD, UK; 2 Mary Lyon Centre, Medical Research Council Harwell, Oxford, Oxfordshire OX11 0RD, UK; 3 Institute of Hepatology, Foundation for Liver Research, 69-75 Chenies Mews, London WC1E 6HX, UK

**Keywords:** Arylformamidase, Kynurenine, Tryptophan, Diabetes, Insulin secretion

## Abstract

Tryptophan metabolites have been linked in observational studies with type 2 diabetes, cognitive disorders, inflammation and immune system regulation. A rate-limiting enzyme in tryptophan conversion is arylformamidase (*Afmid*), and a double knockout of this gene and thymidine kinase (*Tk*) has been reported to cause renal failure and abnormal immune system regulation. In order to further investigate possible links between abnormal tryptophan catabolism and diabetes and to examine the effect of single *Afmid* knockout, we have carried out metabolic phenotyping of an exon 2 *Afmid* gene knockout. These mice exhibit impaired glucose tolerance, although their insulin sensitivity is unchanged in comparison to wild-type animals. This phenotype results from a defect in glucose stimulated insulin secretion and these mice show reduced islet mass with age. No evidence of a renal phenotype was found, suggesting that this published phenotype resulted from loss of *Tk* expression in the double knockout. However, despite specifically removing only exon 2 of *Afmid* in our experiments we also observed some reduction of *Tk* expression, possibly due to a regulatory element in this region. In summary, our findings support a link between abnormal tryptophan metabolism and diabetes and highlight beta cell function for further mechanistic analysis.

## Abstract (precis)

**Summary:** Mice homozygous for a *tm1b* deleted allele have impaired glucose tolerance due to reduced insulin secretion associated with reduced islet mass, but no evidence of renal disease, suggesting a link between abnormal tryptophan metabolism and diabetes.

## INTRODUCTION

Type 2 diabetes (T2D) is a complex disease resulting from the interaction of environmental factors such as obesity, which accounts for 80–85% of the risk, and multiple genetic factors. The disease is characterized by hyperglycemia due to defects in insulin secretion and/or insulin action. Diabetes causes significant mortality and morbidity as a result of complications that develop over prolonged periods of time. Diabetes is a major health problem globally with an estimated 382 million people affected in 2013, and a projected figure of 592 million by 2035 (International Diabetes Federation, 2013; http://www.idf.org/diabetesatlas).

The essential amino acid tryptophan (Trp) is required for protein synthesis and to make the neurotransmitter serotonin. Trp is metabolized in the tryptophan-kynurenine pathway in multiple steps, generating metabolites including kynurenic acid (KYNA), quinolonic acid, xanthurenic acid (XA), and nicotinamide (reviewed Stone and Darlington, 2002).

It has been suggested that dysregulation of XA and kynurenine (KYN) derivatives are involved in insulin resistance and insulin secretion (reviewed Oxenkrug, 2013). XA excretion has been reported to be elevated in the urine of patients with diabetes and in alloxan- and streptozotocin- induced diabetic rats (Hattori et al., 1984; Ikeda and Kotake, 1986). Similarly, a metabolomic analysis in rhesus macaques, comparing normal and spontaneously diabetic animals, identified elevated KYNA in urine as a biomarker of diabetes (Patterson et al., 2011). Further, serum KYN and Trp have been correlated with measures of insulin resistance and beta cell function in patients with hepatitis-C (Oxenkrug et al., 2013). Consistent with these metabolites being biomarkers for diabetes, increased serum KYN, KYNA and 3-hydroxy-KYN has also been associated with diabetic retinopathy (Munipally et al., 2011). Similarly Trp has been found to be elevated in urine of patients with progressive diabetic kidney disease (van der Kloet et al., 2012). Studies *in vitro* have found that metabolites of KYNA and XA inhibit second phase insulin release in rat islets, inhibit insulin biosynthesis and that XA binds to circulating insulin (Kotake et al., 1975; Noto and Okamoto, 1978; Okamoto, 1975; Rogers and Evangelista, 1985).

A number of Trp metabolites have emerged as regulators of the activity of neurotransmitters and have been linked to cognitive disorders (reviewed Stone and Darlington, 2013). Kynurenic acid may act as an endogenous antagonist of glutamate NMDA receptors and as a negative allosteric modulator of nicotinic acetylcholine receptors, and a reduction in KYNA has been shown to improve cognitive function in rodents and non-human primates (Kozak et al., 2014). Consistent with this, KYNA has also been linked with behavioral plasticity in *C. elegans*, with fasting leading to reduced KYNA and activation of NMDA-receptor expressing inter-neurons. This results in activation of a neuropeptide-Y (NPY) like signaling axis, promoting feeding when food becomes available (Lemieux et al., 2015). Increased brain expression of tryptophan 2,3-dioxygenase (TDO), one of the enzymes in the first step of the pathway, has also been linked to Alzheimer's disease in transgenic mice and human tissue (Wu et al., 2013).

Finally, Trp catabolism has been linked with suppression of inflammation and regulation of immunity (Romani et al., 2008; reviewed McGaha et al., 2012).

The enzyme arylformamidase (AFMID, also known as kynurenine formamidase) catalyzes the second step of Trp conversion in the pathway to nicotinic acid, NAD(H), and NADP(H), hydrolyzing N-formyl-L-kynurenine (FKYN) to L-kynurenine (Pabarcus and Casida, 2002, 2005). The *Afmid* and thymidine kinase (*Tk*) genes are adjacent to one another in the genome and transcribed in opposite directions, with their ATG initiation codons only 170 bp apart (Dobrovolsky et al., 2005). Simultaneous inactivation of both *Afmid* and *Tk* genes in mice by deletion of both first exons, results in the elimination of FKYN hydrolysis in the liver and residual (13%) hydrolysis in the kidney (Dobrovolsky et al., 2005). In addition, plasma concentrations of Trp are unaltered but FKYN, KYN and KYNA are elevated, also liver and kidney levels of nicotinamide derivatives remain the same, indicating that the KYN pathway is disturbed but functional in double knockout mice (Dobrovolsky et al., 2005). Double knockout mice also exhibit kidney failure and an abnormal immune system with mice usually dying before 6 months of age (Dobrovolsky et al., 2005, 2003).

In order to further investigate the functional consequences, for type 2 diabetes, of loss of AFMID *in vivo,* we have characterized an exon 2 knockout of the *Afmid* gene using the EUCOMM allele *Afmid^tm1b(EUCOMM)Wtsi^* (Skarnes et al., 2011), available through the International Mouse Phenotyping Consortium (IMPC, https://www.mousephenotype.org/).

We have found that mice homozygous for the tm1b deleted allele have impaired glucose tolerance due to reduced insulin secretion associated with reduced islet mass. There was no evidence of renal disease in these *Afmid^tm1b/tm1b^* mice, unlike the double *Afmid* and *Tk* knockout, suggesting that the renal traits are due to complete loss of *Tk*. This study further implicates KYN pathway metabolites in diabetes.

## RESULTS

Initial data from limited numbers of *Afmid* Tm1a allele mice indicated that they had a strong diabetes phenotype, for example six males around 21 weeks of age had free fed blood glucose concentrations ranging from ∼29 to 52 mM (data not shown). Based on these data we decided to carry out comprehensive phenotyping to fully characterize the phenotype and understand its causes. The Tm1a allele is a gene trap expressing LacZ and contains a selectable neomycin cassette; therefore we decided to convert it to a full exon 2 deletion allele carrying only the LacZ reporter. This was achieved by using a β-actin Cre recombinase giving germline deletion. The Cre was then bred out before generating the animals for phenotyping.

**Fig. 1.**
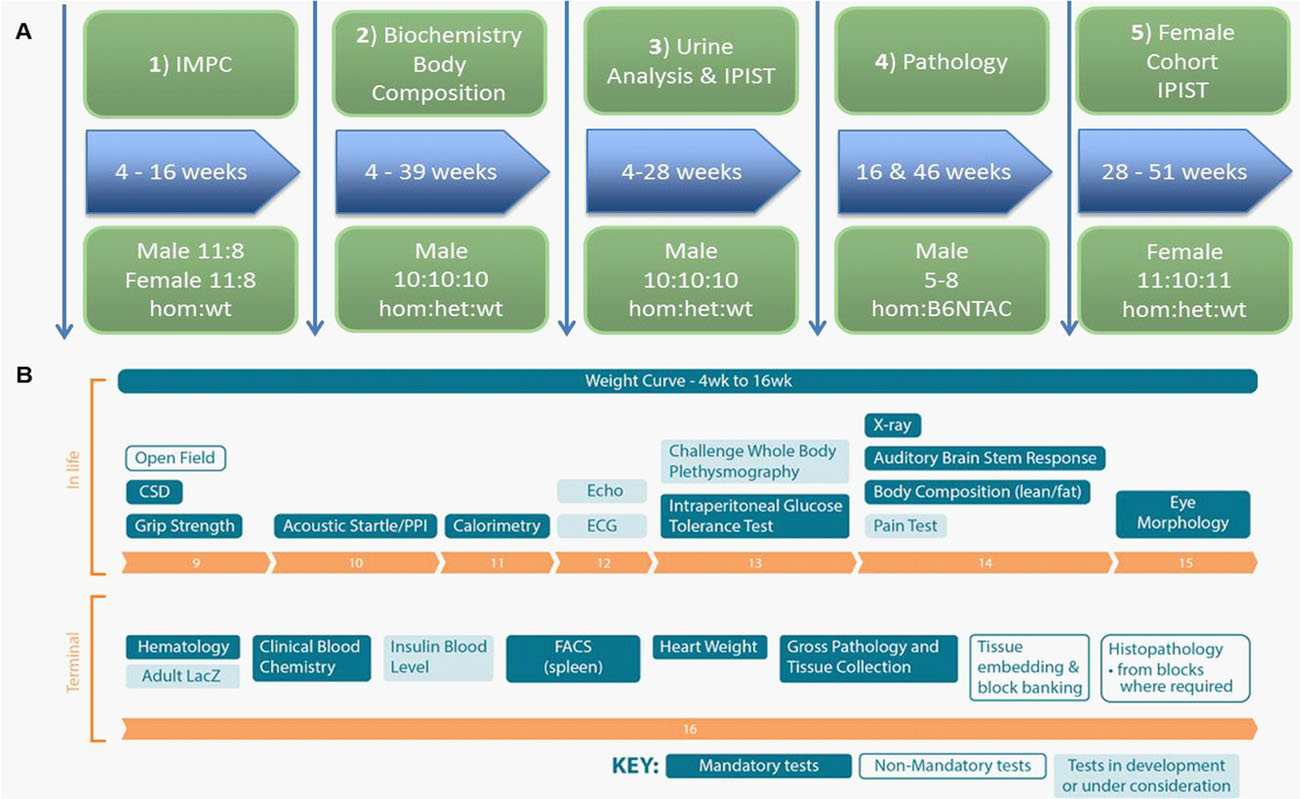
**Overview of five phenotyping cohorts.** (A) A cohort of 11 male and 8 female *Afmid^tm1b/tm1b^* mice on a C57BL6/NTac background were bred for the IMPC phenotyping pipeline (1). Two further cohorts of 10 males for each *Afmid^tm1b/tm1b^*, *Afmid^tm1b/+^* and *Afmid^+/+^* genotype were bred for blood based tests and one for urine analysis (2,3). A smaller cohort of 5–9 males of each genotype was also bred for pathology analysis (4). Finally a cohort of 11 *Afmid^tm1b/tm1b^* and *Afmid^+/+^* and 10 *Afmid^tm1b/+^* females were generated to investigate developing glucose phenotypes in older mice (5). (B) A schematic of the IMPC pipeline phenotyping timeline. wt=*Afmid^+/+^*, het=*Afmid^tm1b/+^*, hom*=Afmid^tm1b/tm1b^*.

Five cohorts were bred for separate dedicated phenotyping experiments. A cohort of 11 male and 8 female homozygous knockout (*Afmid^tm1b/tm1b^*) mice on a C57BL6/NTac background were bred for the IMPC phenotyping pipeline (http://www.mousephenotype.org/impress) (Fig. 1A,B). Two further cohorts of 10 males for each homozygous, heterozygous (*Afmid^tm1b/+^*) and wild-type (*Afmid^+/+^*) genotype were bred, one for longitudinal blood based tests and one for urine analysis (Fig. 1A). A smaller cohort of males of each genotype were also bred for pathology analysis at varying time points (16 weeks, *n*=5 *Afmid^+/+^* and *n*=7 *Afmid^tm1b/tm1b^*; 46 weeks, *n*=8 *Afmid^+/+^* and *n*=9 *Afmid^tm1b/tm1b^*; Fig. 1A). Finally a cohort of 11 *Afmid^tm1b/tm1b^* and 11 *Afmid^+/+^* and 10 *Afmid^tm1b/+^* females were generated to investigate developing glucose phenotypes in older female mice (Fig. 1A).

### Expression of *Afmid* and *Tk* in total RNA

**Fig. 2.**
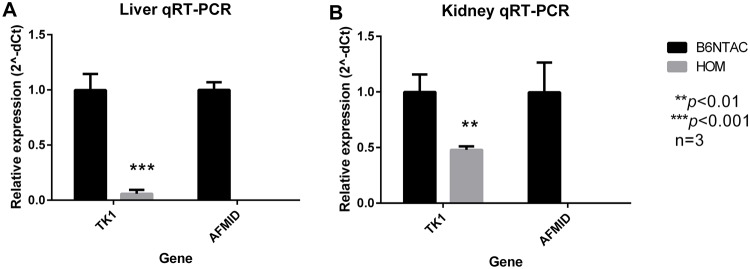
**TK1 and AFMID expression.** qRT-PCR relative expression data for thymidine kinase 1 (TK1; Mm01246403_g1) and arylformamidase (AFMID; Mm00510775_m1) in liver (A) and kidney (B) samples taken from male *Afmid^tm1b/tm1b^* mice and C57BL6/NTac controls. These results confirm complete silencing of *Afmid* in *Afmid^tm1b/tm1b^* mice, however, TK1 was also reduced. Values are expressed as mean±s.d. (*n*=3/group). **P>0.01, ***P>0.001 as compared to C57BL6/NTac controls (Student's 2-tailed *t*-test). HOM*=Afmid^tm1b/tm1b^*.

In order to verify loss of expression of the *Afmid* gene transcript in *Afmid^tm1b/tm1b^* knockout animals and to establish whether the exon 2 deletion affected expression of the immediately adjacent *Tk* gene, we carried out qRT-PCR on liver and kidney total RNA. The expression of *Afmid* was undetectable in both liver and kidney of homozygous mice. Surprisingly, the expression of *Tk* was also reduced by 52% in the kidney and 94% in the liver (Fig. 2). There was no detectable expression of *Afmid* or *Tk* in RNA from isolated islets of wild-type or mutant mice.

### *Afmid^tm1b/tm1b^* mice show impaired glucose tolerance

**Fig. 3.**
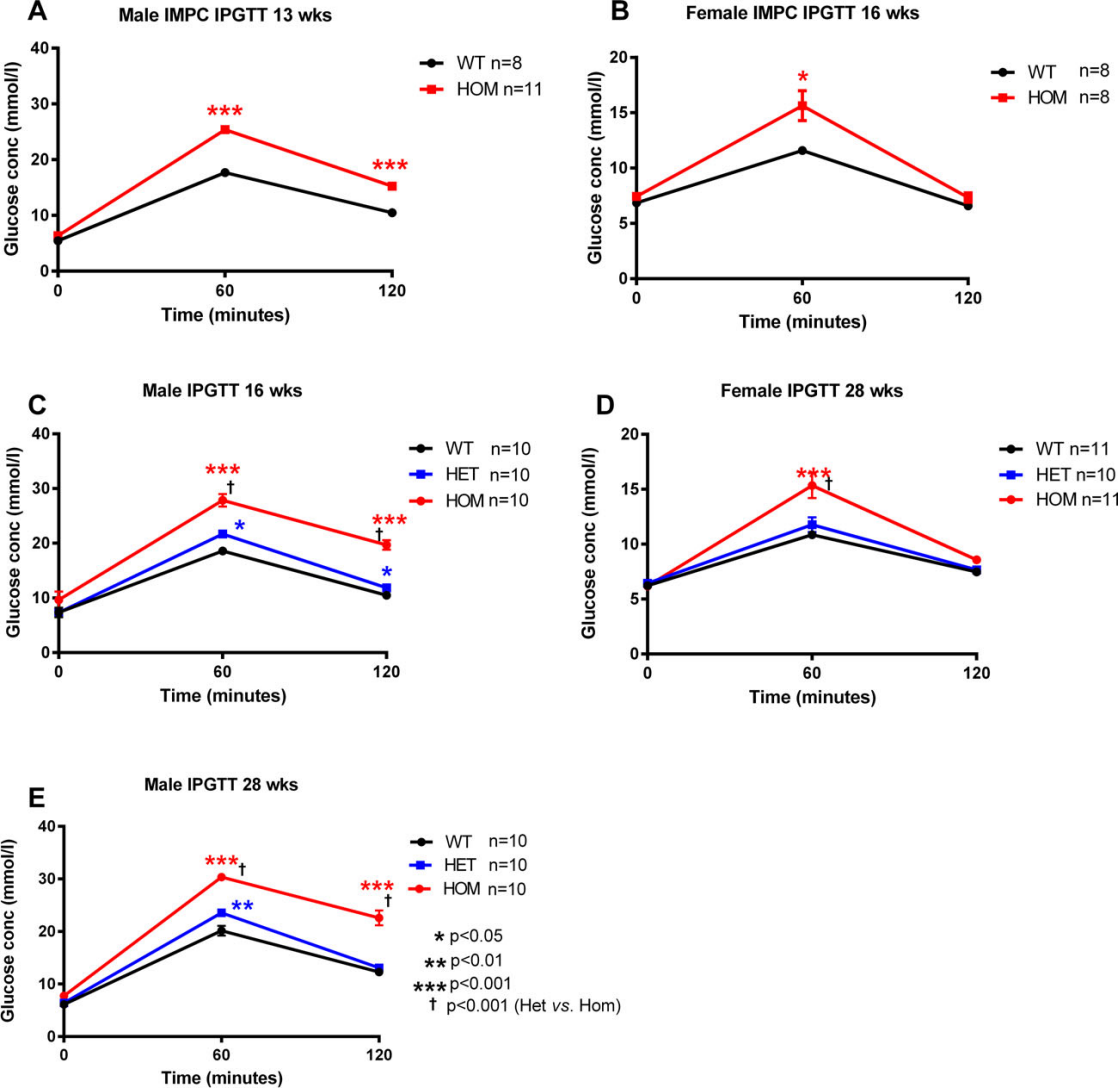
**Glucose tolerance test.** Intraperitoneal glucose tolerance test (IPGTT) data for male and female *Afmid^tm1b/tm1b^* mice and *Afmid^tm1b/+^* and *Afmid^+/+^* controls. Glucose tolerance was measured during an IPGTT at 13 (A), 16 (C) and 28 (E) weeks for male mice and 16 (B) and 28 (D) weeks for female mice. The results show that both male and female *Afmid^tm1b/tm1b^* mice demonstrate impaired glucose tolerance at all time points. Values are expressed as mean±s.e.m. (*n*=8–11/group). *P>0.05, **P>0.01, ***P>0.001 as compared to *Afmid^+/+^* controls; †*P*>0.001 as compared to *Afmid^+/tm1b^* (2-way ANOVA with repeated measures and Bonferroni correction). WT=*Afmid^+/+^,* HET=*Afmid^tm1b/+^*, HOM*=Afmid^tm1b/tm1b^*.

Glucose tolerance was measured during an IPGTT at 13 (IMPC cohort 1, Fig. 1), 16 and 28 weeks (cohort 2, Fig. 1) for male mice and 16 (IMPC cohort 1, Fig. 1) and 28 weeks for female mice (cohort 5, Fig. 1). *Afmid^tm1b/tm1b^* mice exhibited impaired glucose tolerance at all ages tested (Fig. 3A-E). Glucose levels in male *Afmid^tm1b/tm1b^* mice were significantly elevated compared to *Afmid^tm1b/+^* and *Afmid^+/+^*controls at 60 min post-glucose load and failed to return to normal levels after 120 min, becoming progressively worse with age (Fig. 3A,C,E). *Afmid^tm1b/+^* heterozygous male mice were tested at 16 and 28 weeks and exhibited mild impaired glucose tolerance with significantly elevated glucose levels at 60 and 120 min at 16 weeks and at 60 min at 28 weeks compared to *Afmid^+/+^* controls (Fig. 3C,E). Female *Afmid^tm1b/tm1b^* mice showed significantly elevated glucose levels 60 min post-glucose load at both 16 and 28 weeks when compared to controls but levels returned to normal after 120 min (Fig. 3B,D). *Afmid^tm1b/+^* heterozygous female mice were not significantly different to controls (Fig. 3D).

**Fig. 4.**
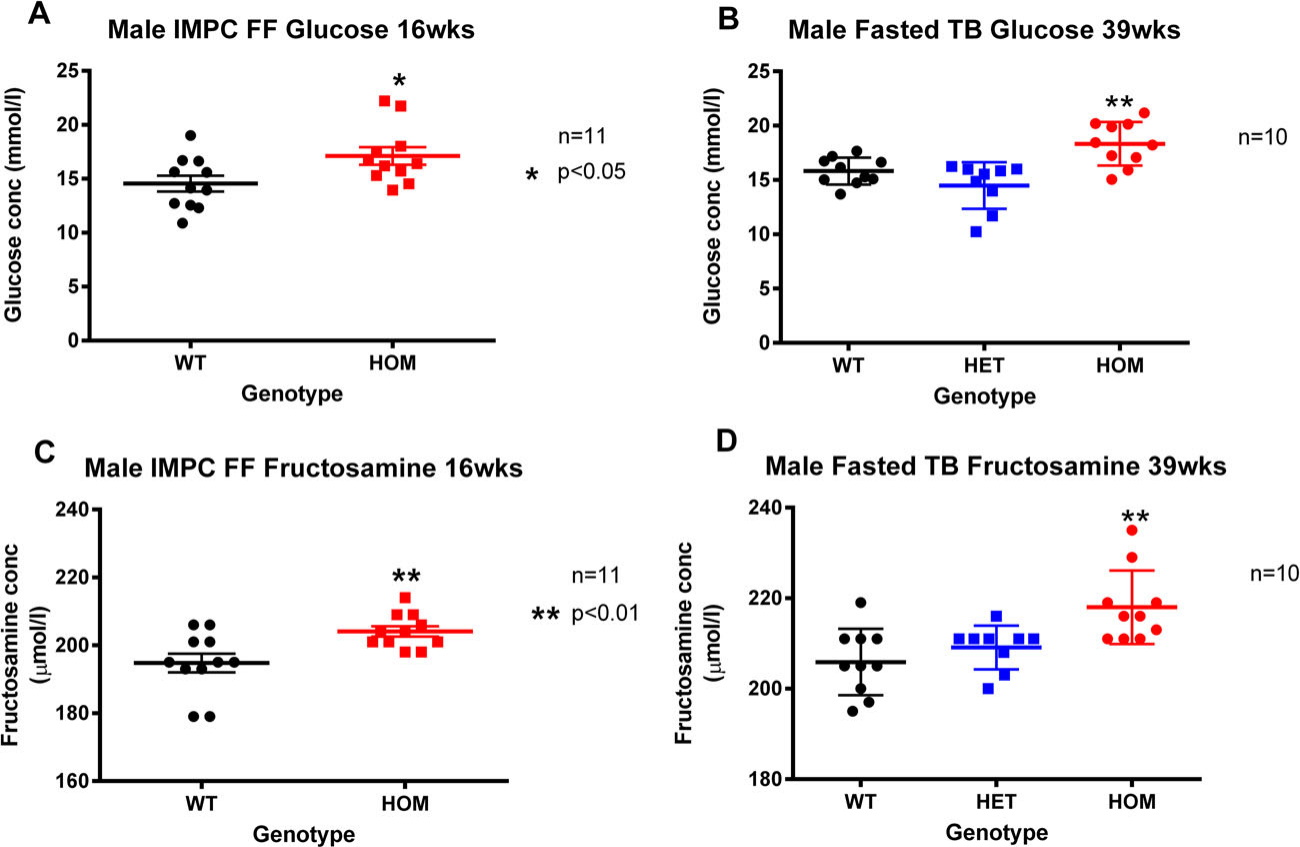
**Glucose and fructosamine clinical chemistry.** Clinical chemistry parameters were measured at 16 (free-fed; A,C) and 39 (fasted; B,D) weeks of age in male *Afmid^tm1b/tm1b^* mice and *Afmid^tm1b/+^* and *Afmid^+/+^* controls. Glucose and fructosamine levels were significantly elevated at both 16 and 39 weeks. Values are expressed as mean±s.e.m. (*n*=10–11/group). *P>0.05, **P>0.01 as compared to *Afmid^+/+^* controls (1-way ANOVA with Bonferroni correction). WT=*Afmid^+/+^*, HET=*Afmid^tm1b/+^*, HOM*=Afmid^tm1b/tm1b^*, FF=Free Fed, TB=Terminal Bleed.

Clinical chemistry confirmed significant elevations in plasma glucose and fructosamine (an indicator of glycaemic status during the preceding 1–2 weeks) in cohorts of male *Afmid^tm1b/tm1b^* mice respectively aged 16 and 39 weeks (Fig. 4A-D). These trends were not observed in 16 and 51 week old female *Afmid^tm1b/tm1b^* mice (data not shown).

### Male *Afmid^tm1b/tm1b^* mice show impaired insulin secretion

**Fig. 5.**
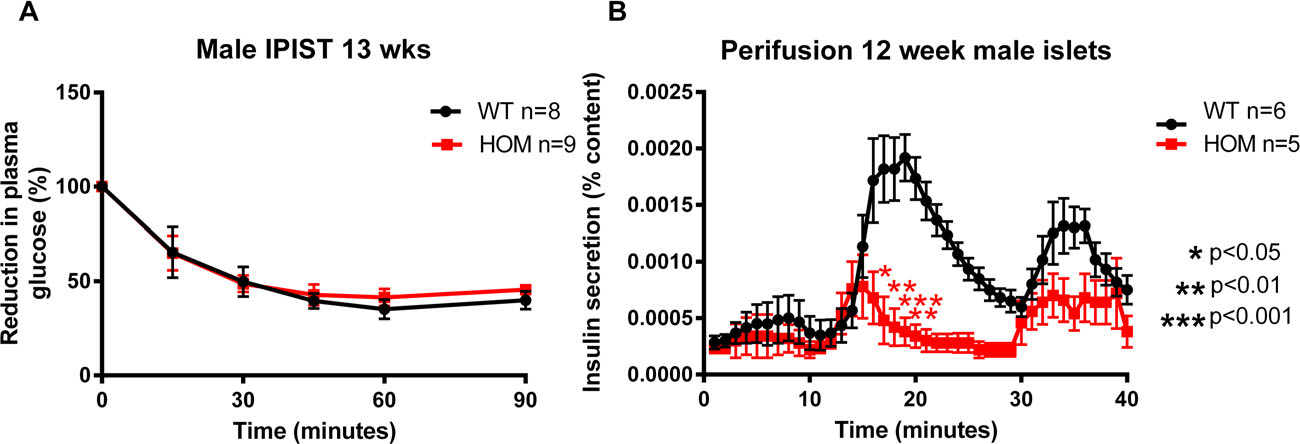
**Insulin tolerance test and islet perifusion.** (A) Intraperitoneal insulin sensitivity test (IPIST) data for male *Afmid^tm1b/tm1b^* mice and *Afmid^+/+^* controls at 13 weeks of age. (B) Pancreatic islet perifusion normalized insulin secretion data for male *Afmid^tm1b/tm1b^* mice and *Afmid^+/+^* controls at 12 weeks of age. There were no differences in insulin tolerance, however *Afmid^tm1b/tm1b^* mice demonstrated reduced insulin secretion from their pancreatic islets. Values are expressed as mean±s.e.m. (*n*=5–6/group). *P>0.05, **P>0.01, ***P>0.001 as compared to *Afmid^+/+^* controls (2-way ANOVA with repeated measures and Bonferroni correction). WT=*Afmid^+/+^*, HOM*=Afmid^tm1b/tm1b^*.

To investigate why *Afmid^tm1b/tm1b^* male mice are glucose intolerant we first carried out an intraperitoneal insulin sensitivity test (IPIST) at 13 weeks of age (cohort 3, Fig. 1), (paralleling the IMPC cohort IPGTTs that showed impaired glucose tolerance at this age) to evaluate whether the mice were insulin resistant (Fig. 5A). Reduction in blood glucose concentrations during the test was not significantly different between *Afmid^tm1b/tm1b^* and *Afmid^+/+^* mice indicating that they had similar insulin sensitivities. Therefore, we hypothesized that the differences in glucose tolerance may be explained by a defect in insulin secretion. In order to test this, we measured glucose stimulated insulin secretion by collecting 50 isolated islets of similar size from individual male *Afmid^tm1b/tm1b^* and *Afmid^+/+^* mice and perifusing them with glucose at 2 mM (basal), then 20 mM, and then finally at 20 mM with the addition of 500 μM tolbutamide (Fig. 5B). Pancreatic islets were isolated at 12 weeks of age from *Afmid^tm1b/tm1b^* and *Afmid^+/+^* mice and strikingly, qualitatively an approximate 50% reduction was observed in the *Afmid^tm1b/tm1b^* digests (data not shown). There was no difference in basal insulin secretion between *Afmid^tm1b/tm1b^* and *Afmid^+/+^* islets, however in response to 20 mM glucose, there was significantly (*P*<0.001) less insulin secreted in *Afmid^tm1b/tm1b^* islets (Fig. 5B). The *Afmid^tm1b/tm1b^* islets showed a trend of secreting less insulin in response to 500 μM tolbutamide plus 20 mM glucose compared to *Afmid^+/+^* islets (Fig. 5B). There was no significant difference in total insulin content between the collection of *Afmid^tm1b/tm1b^* (mean 1576.1±138.0 pg/ml) and *Afmid^+/+^* (mean 3098.7±518.5 pg/ml) islets, although there was a clear trend towards reduced content in the *Afmid^tm1b/tm1b^*.

### Male *Afmid^tm1b/tm1b^* mice form normal islets but islet mass decreases with age

**Fig. 6.**
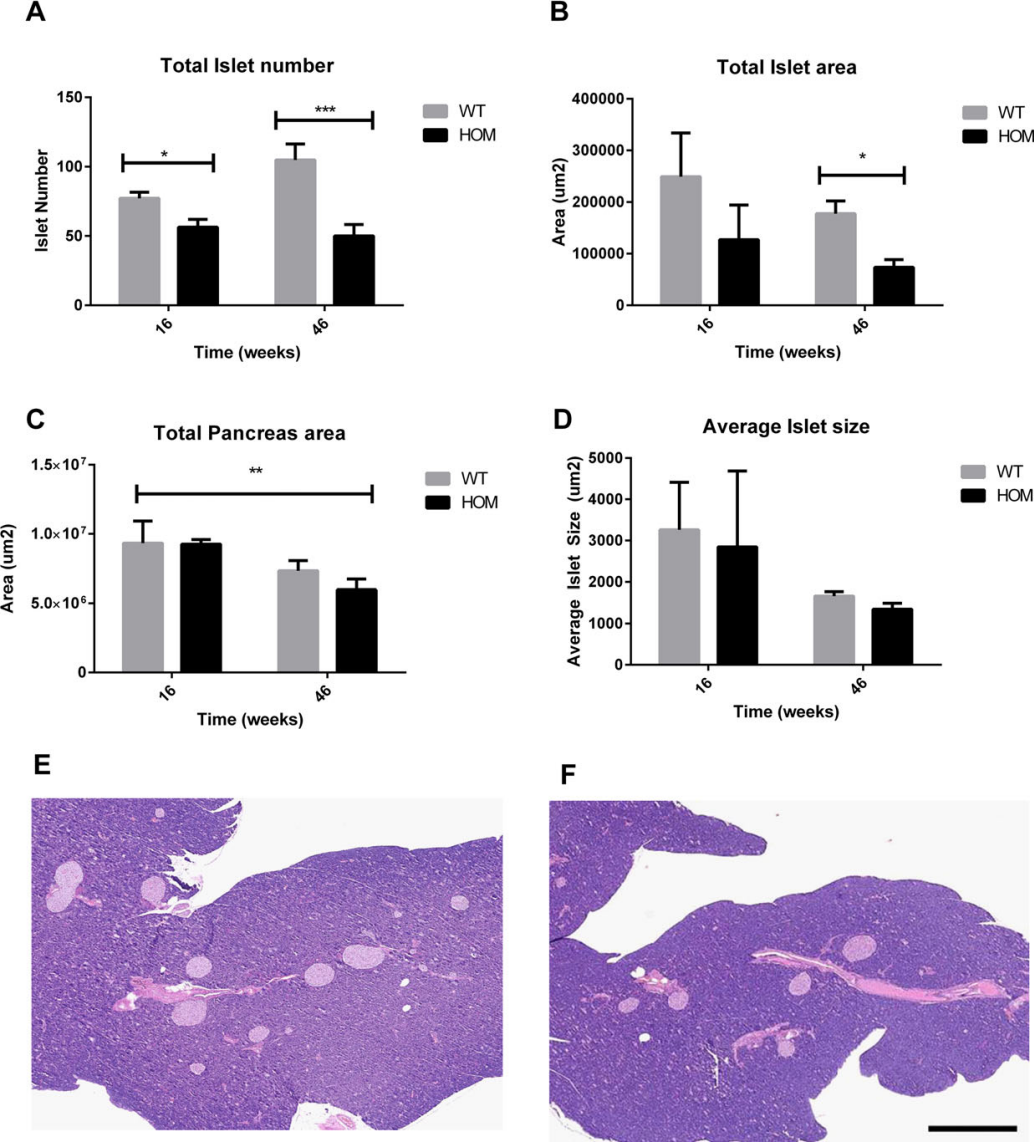
**Islet histology.** (A-D) Total islet number (A), total islet area (B), total pancreas area (C) and average islet size (D) as analyzed from whole pancreas sections taken from male *Afmid^tm1b/tm1b^* mice and *Afmid^+/+^* controls at 16 and 46 weeks of age. (E,F) Representative Hematoxylin & Eosin stained sections from *Afmid^+/+^* (E) and *Afmid^tm1b/tm1b^* (F) mice at 46 weeks of age (scale bar=250 µm). All islets within a section were counted, and a representative five sections were sampled from throughout the pancreas each 200 µm apart. *Afmid^tm1b/tm1b^* mice had reduced islet number at both time points and a reduced total islet area at 46 weeks old. Values are expressed as mean±s.e.m. (*n*=5–9/group). *P>0.01 as compared to *Afmid^+/+^* controls (2-way ANOVA). WT=*Afmid^+/+^*, HOM*=Afmid^tm1b/tm1b^*.

Given the impaired glucose stimulated insulin secretion and qualitative differences in the number of islets isolated, we next evaluated islet mass by histological methods. Glucagon and insulin immuno-staining of pancreas serial sections showed no qualitative difference in islet architecture in terms of numbers or arrangement of α and β cells between male *Afmid^tm1b/tm1b^* and *Afmid^+/+^* mice at 16 and 46 weeks of age (data not shown). Islet number and islet area were then quantified to look for differences (Fig. 6A,B). Analysis of pancreatic immunohistochemistry revealed that by 16 weeks of age there was a significant reduction in islet number in *Afmid^tm1b/tm1b^* in comparison to the *Afmid^+/+^* controls (*P*=0.0156) despite there being no differences in the total area of the pancreas (*P*=0.9653) (Fig. 6A,C). Analysis by 2-way ANOVA showed that the total pancreatic area decreased over time in both groups (*P*=0.0052) (Fig. 6C). By 46 weeks of age total islet number and total islet area were both significantly reduced in the *Afmid^tm1b/tm1b^* mice compared to *Afmid^+/+^* (*P*=0.002 and 0.004 respectively, Fig. 6A,B; representative images from 46 weeks are presented in Fig. 6E,F). Despite these reductions the average islet size remained the same for both groups (Fig. 6D).

### No histopathology was observed in *Afmid^tm1b/tm1b^* kidney or liver

No abnormalities were detected during the IMPC histopathological investigation of 32 tissues at 16 weeks (data not shown). Given the reported renal phenotypes in double knockout mice additional kidney and liver sections were also examined at 28 weeks, but no histopathology was observed (data not shown).

### No differences were observed in body mass or composition

There were no significant differences in weight or percentage fat and lean mass between *Afmid^+/+^*, *Afmid^tm1b/+^* or *Afmid^tm1b/tm1b^* mice, for males or females (data not shown).

### Kynurenic and Xanthurenic acid detected by NMR spectroscopy in *Afmid^tm1b/tm1b^* urine

**Fig. 7.**
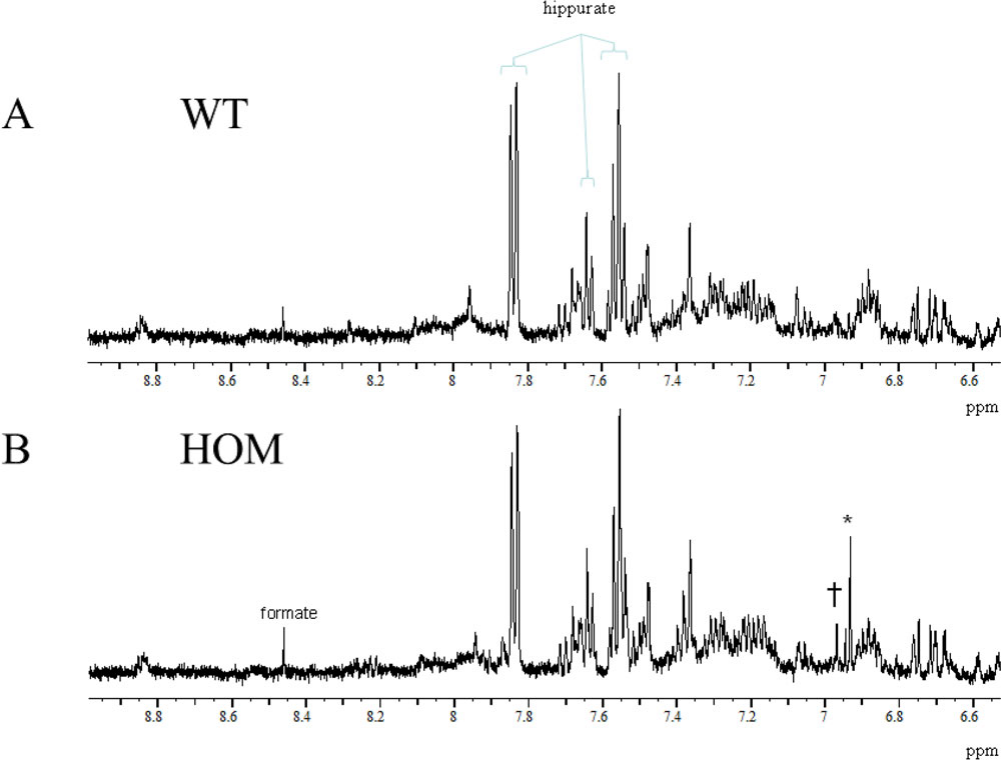
**Representative urinary ^1^H NMR spectra.** Urinary ^1^H NMR spectra from an *Afmid^+/+^* control mouse (A) and an *Afmid^tm1b/tm1b^* mouse (B) showing the aromatic region of the spectrum. Peaks are assigned to formate and hippurate in both data sets, but additionally to kynurenic acid (*) and xanthurenic acid (†) in the urinary ^1^H NMR spectrum from the *Afmid^tm1b/tm1b^* mouse. WT=*Afmid^+/+^*, HOM*=Afmid^tm1b/tm1b^*.

As the *Afmid* gene encodes a key enzyme in the kynurenine pathway we evaluated pathway metabolites in urine samples in a longitudinal study. The urinary NMR spectra of *Afmid^tm1b/tm1b^* mice showed additional small resonances in the aromatic region, which were assigned to KYNA and XA (Fig. 7). These peaks were detected from 6 weeks of age, became more prominent with increasing age and were absent in *Afmid^+/+^* mice.

## DISCUSSION

Initial phenotype data from *Afmid* tm1a mice indicated overt diabetes (very high blood glucose, polydipsia and polyuria), however, detailed phenotyping of tm1b (exon 2 deletion) mice revealed a less severe glucose intolerance phenotype. The reason for this difference is unknown although we can speculate that the tm1a gene trap allele with LacZ and neo cassettes upstream of exon 2 disrupts an element within the intron that affects expression of another gene or genes. Supporting this, we found that expression of the closely adjacent *Tk* gene in homozygous tm1b *Afmid* mice was approximately 50% reduced in kidney and about 90% reduced in liver, indicating that regulation of this gene was disrupted by deleting exon 2 of *Afmid* and replacing it with a LacZ reporter. Previous reports of fatal kidney disease in *Afmid* and *Tk* double knockouts is in contrast to *Afmid* tm1b mice that did not exhibit any renal phenotypes (Dobrovolsky et al., 2005, 2003). Given that both models lack *Afmid* expression it is likely that the renal phenotype in double knockout mice is due to a lack of TK activity and that our model retains sufficient TK activity to be healthy. Alternatively, defects in both genes could act synergistically to cause disease in sensitive tissues or a potential developmental defect. Thymidine kinase is required for thymidine nucleotide salvage for use in DNA synthesis and repair, whilst the *Afmid* block may affect the first rate-limiting step of tryptophan degradation that is superoxide consuming and generates formyl kynurenine for conversion by AFMID (Stone and Darlington, 2002). A testable hypothesis would be that *Afmid* knockout results in disruption of the kynurenine pathway leading to DNA damage, possibly by superoxide, and in the case of *Tk* deficiencies abnormalities in repair that lead to cellular dysfunction in susceptible tissues and cells.

The male *Afmid^tm1b/tm1b^* mice retain normal insulin sensitivity and the impaired glucose tolerance is likely caused by a defect in both first and second phase insulin secretion as measured in isolated perifused islets. The trend for reduced tolbutamide induced insulin secretion suggests the defect is downstream of the K_ATP_ channel as this agent closes the channel and should stimulate secretion. The lack of impaired glucose tolerance in *Afmid^tm1b/tm1b^* female mice may be due to a protective hormonal affect. Histologically we also observed loss of islet number in male *Afmid^tm1b/tm1b^* mice in comparison with *Afmid^+/+^* littermates. Histological analysis of the double knockout pancreas has similarly been reported to be deficient in islets (Dobrovolsky et al., 2003). Consistent with an insulin secretory defect a number of studies have linked KYN metabolites with impaired insulin secretion *in vitro* (Noto and Okamoto, 1978; Okamoto, 1975; Rogers and Evangelista, 1985). Furthermore, that xanthurenic acid binds to circulating insulin and prevents the hormone binding to cells, although we have not observed insulin resistance in our model (Kotake et al., 1975). NMR analysis of urine showed the presence of both kynurenic acid and xanthurenic acid in *Afmid^tm1b/tm1b^* mice and therefore it is possible that the loss of islet number may be due to a toxicity effect halting development of these particular cells rather than apoptosis as there was no morphological evidence of cell death. Alternatively, KYN metabolites may exert a physiological effect on islet cells suppressing insulin secretion. We did not detect either *Afmid* or *Tk* expression in isolated islets of wild-type or mutant animals (data not shown), indicating that the islet defect is not intrinsic to islet cells.

Kynurenic acid has also been linked with behavioral plasticity (Lemieux et al., 2015) and increased brain expression of tryptophan is linked to Alzheimer's disease (Wu et al., 2013). Therefore a small cognition screen consisting of open field habituation and fear conditioning was carried out on male *Afmid^tm1b/tm1b^* mice and *Afmid^+/+^* controls. There were no observed differences in the fear conditioning studies, however, the open field habituation demonstrated differences in activity level which were slightly increased in *Afmid^tm1b/tm1b^* mice (data not shown). This effect was not borne out in the learning paradigm as the *Afmid^tm1b/tm1b^* mice still showed reduced activity on day two of testing.

Both KYNA and XA are downstream of the conversion of formyl kynurenine to L-kynurenine catalyzed by AFMID. It is therefore unexpected that their level of excretion increases when this gene is knocked out, however, elevation of these metabolites in plasma was also observed in the double knockout mice (Dobrovolsky et al., 2005). Thus there appears to be alternative pathways to generate these metabolites from formyl kynurenine.

In summary, we report that loss of *Afmid* and a reduction in *Tk* expression results in defective glucose stimulated insulin secretion and impaired glucose tolerance. In order to further dissect the function of these two genes more precisely it would be useful to study the effects of mutations, such as a stop codon, using CRISPR/Cas9 technology that would not disrupt intronic elements or their spacing, but eliminate gene function. Our studies provide further support of an association between kynurenine metabolites and diabetes (Hattori et al., 1984; Ikeda and Kotake, 1986; Kotake et al., 1975; Munipally et al., 2011; Noto and Okamoto, 1978; Okamoto, 1975; Oxenkrug, 2013; Oxenkrug et al., 2013; Patterson et al., 2011; Rogers and Evangelista, 1985; van der Kloet et al., 2012).

## MATERIALS AND METHODS

### Animal husbandry

Mice were kept and studied in accordance with UK Home Office legislation and local ethical guidelines issued by the Medical Research Council (Responsibility in the Use of Animals for Medical Research, July 1993; home office license 30/3146). Mice were kept under controlled light (light 7am–7pm, dark 7pm–7am), temperature (21±2°C) and humidity (55±10%) conditions. They had free access to water (9–13 ppm chlorine) and were fed *ad libitum* on a commercial diet (SDS Rat and Mouse No. 3 Breeding diet, RM3). Tm1a mice were derived from C57BL6/NTac ES cells as previously described (Bradley et al., 2012; Pettitt et al., 2009; Skarnes et al., 2011). Mice were bred to a Cre-recombinase line (C57BL/6NTac-Tg(ACTB-cre)^3Mrt^/H) to delete the selection cassette and exon 2 of the *Afmid* gene, generating the null allele *Afmid^tm1b^*, on a C57BL/6NTac background. After breeding out the Cre-recombinase, male and female *Afmid^tm1b/tm1b^* and *Afmid^+/+^* mice were phenotyped via the IMPC pipeline at the MRC Harwell. Additional cohorts of male and female *Afmid^tm1b/tm1b^*, *Afmid^tm1b/+^* and *Afmid^+/+^* mice were bred for alternative phenotyping tests (Fig. 1).

### Experimental design

Five cohorts were bred for separate dedicated phenotyping experiments. Cohort sizes were chosen based on previous experience and power calculations using data from tests applied on other genetic mouse models of impaired glucose tolerance. A cohort of 11 male and 8 female *Afmid^tm1b/tm1b^* mice on a C57BL6/NTac background were bred for the IMPC phenotyping pipeline (http://www.mousephenotype.org/impress). Two further cohorts of 10 males for each *Afmid^tm1b/tm1b^*, *Afmid^tm1b/+^* and *Afmid^+/+^* genotype were bred, one for longitudinal blood based tests and one for urine analysis. Smaller cohorts of 6 males of each genotype were also bred for pathology analysis. Finally a cohort of 11 *Afmid^tm1b/tm1b^* and *Afmid^+/+^* and 10 *Afmid^tm1b/+^* females were generated to investigate developing glucose phenotypes in older mice (Fig. 1).

### Genotyping

Mice were genotyped using Taqman probe copy count qPCR. The qPCR assays for wild-type or LacZ (marking the knockout allele), were FAM labelled and run in duplex on a TaqMan system based on real-time detection of accumulated fluorescence (ABI Prism 7900, Applied Biosystems) with a VIC labelled internal control dot1l. Results were analyzed and copy counted using the ABI CopyCaller software v.2 (ABI). Ct values of greater than 30 were called as a fail. Each sample was run with a technical duplicate and each assay run with 7 controls of known genotype and a blank control.

### Quantitative RT-PCR

RNA was extracted from snap frozen tissues using an RNeasy Mini Kit (Qiagen, UK) according to the manufacturer's instructions. cDNA was generated using Superscript III enzyme (Invitrogen, UK) and analyzed by quantitative RT-PCR using the TaqMan system based on real-time detection of accumulated fluorescence (ABI Prism 7700, Perkin-Elmer Inc., USA). To select the appropriate house-keeping genes geNorm analysis using 12 reference genes (Primerdesign, UK) was undertaken against all samples. Samples were tested in triplicate and gene expression normalized relative to the expression of house-keeping genes *18S* (Mm02601777_g1) and *Gapdh* (Mm99999915_g1) for liver and *ActB* (Mm00607939_s1) and *Rpl13a* (MM02601777_g1) for kidney. *Afmid* (Mm00510775_m1) and *Tk1* (Mm01246403_g1) Fam-labeled Taqman probes were purchased from Life Technologies (ABI, USA).

### Intraperitoneal glucose tolerance test (IPGTT)

IPGTT's were carried out according to the IMPReSS protocols (https://www.mousephenotype.org/impress/protocol/87/8) using 2 g glucose per kg body weight. Mice were fasted overnight and blood sampled under a local anesthetic at 0 min (baseline) and 60 and 120 min post glucose injection. Whole blood glucose was measured using an AlphaTRAK meter and test strips (Abbott Animal Health, UK). At the 13 week time point male plasma samples were also run on a GM9 glucose analyzer (Analox Instruments, UK) which is more sensitive than the AlphaTRAK meter. Plasma insulin was assayed using a Mouse insulin ELISA kit (Mercodia, Sweden).

### Intraperitoneal insulin sensitivity test (IPIST)

Animals were fasted for 4 h and blood sampled under a local anesthetic at 0 min (baseline), prior to injection of 1 IU insulin per kg of body weight, and subsequently at 15, 30, 45, 60 and 90 min. Whole blood glucose was measured using an AlphaTRAK meter and test strips (Abbott Animal Health).

### Free fed bleeds

The test was undertaken at the same time of day (morning) and blood sampled from the lateral tail vein under local anesthetic. Whole blood glucose was measured using an AlphaTRAK meter and test strips (Abbott Animal Health). Plasma insulin was assayed using a Mouse insulin ELISA kit (Mercodia).

### Clinical biochemistry

Terminal blood samples were collected from male mice aged 16, 39 and female mice aged 51 weeks. 16 week old mice were free-fed prior to terminal blood collections while 39 and 51 week old mice were fasted overnight (max. 18 h). Blood samples were collected under terminal isofluorane inhalation anesthesia by retro-orbital puncture into both pediatric EDTA and lithium heparin coated tubes. EDTA whole blood samples for hematology were placed on a rotary mixer for 30 min immediately after collection and kept at room temperature until a full blood count and differential analysis was performed on board a Siemens Advia 2120 hematology analyzer using settings and reagents recommended by the manufacturer. Lithium heparin samples were kept on wet ice until being centrifuged for 10 min at 8000×***g*** in a centrifuge set to room temperature. The resulting plasma was analyzed on board a Beckman Coulter AU680 clinical chemistry analyzer using reagents and settings recommended by the manufacturer.

### Histology

The IMPC histopathology screen at 16 weeks involved fixing, processing and embedding in wax, sectioning and staining with Hematoxylin and Eosin (H&E) 32 tissues (https://www.mousephenotype.org/impress/protocol/99/7), including the liver, kidney and pancreas which were assessed by a pathologist . For additional cohorts of mice the kidneys and livers were collected and analyzed in a similar way at 16 and 46 weeks of age. The pancreas, with the spleen attached, was fixed in neutral buffered formaldehyde and mounted in wax longitudinally using the spleen as an anchor in LS orientation. Serial sections 5 μm thick were cut 40 μm apart through the whole pancreas and either left unstained for immunohistochemistry or stained with H&E. To calculate a representative islet area five H&E-stained sections 200 μm apart, starting at a depth of 150 μm, from five to nine mice of each genotype, were photographed and the islet and total pancreas area calculated using ImageJ64.

### Islet immunohistochemistry

Immunohistochemistry was carried out on paraffin serial sections of the pancreas at 5, 16, 28, and 46 weeks. The following primary antibodies were used; rabbit anti-Insulin (1:100, Santa Cruz) and rabbit anti-Glucagon (1:1000, Millipore). A goat anti-rabbit secondary (1:250, Jackson) was used for both primary antibodies. Sections were counter-stained with Gill's formulation #3 hematoxylin. A rabbit IgG antibody was used as a negative control.

### Islet perifusion

Mice were killed by cervical dislocation and islets isolated by liberase digestion and handpicking as previously described (Shimomura et al., 2009). Islets were maintained in RPMI 1640 containing 11 mM glucose (Gibco) supplemented with 10% FBS and 100 U/ml penicillin and 100 µg/ml streptomycin and cultured for 24 h at 37°C in humidified 5% CO_2_ in air, before experimentation. Insulin secretion was measured from 50 islets of equal size for each genotype. Islets were perifused at 37°C and a rate of 1 ml per min with Krebs-Ringer-HEPES buffer (in mM): 140 NaCl, 3.6 KCl, 2.6 CaCl_2_, 0.5 NaH_2_PO_4_, 0.5 MgSO_4_7H_2_O, 5 HEPES, and 2 NaHCO_3_, pH 7.4 (with NaOH), plus 0.2% BSA and glucose and tolbutamide as indicated. A 500 mM stock solution of tolbutamide was made in DMSO and diluted as required. After 30 min pre-perifusion in 2 mM glucose, the perifusate was collected every 1 min for 10 min in 2 mM glucose, 20 min in 20 mM glucose and finally 10 min in 20 mM glucose plus 500 μM tolbutamide. Finally islets were incubated overnight at −20°C with acidified ethanol solution (95% ethanol, 5% acetic acid) to extract all insulin. Insulin was measured using a Rat/Mouse Insulin ELISA kit (Millipore, UK) and normalized to insulin content.

### Body mass and composition

Body mass was measured every 2 weeks using scales calibrated to 0.01 g. Analysis of body composition was performed using an Echo MRI whole body composition analyzer (Echo Medical System, USA) every 4 weeks for males and at 35, 38 and 42 weeks for females.

### Urinary NMR studies

Urine samples were collected at the same time of day from male *Afmid^tm1b/tm1b^* and *Afmid^+/+^* mice every 2 weeks from 6 to 12 weeks and then every month until 28 weeks of age. Free catch urine was collected into a sterile microcentrifuge tube that was placed at 4°C for a maximum of 1 h before centrifugation at 16,060 ***g*** for 1 min, the supernatant was removed and stored at −20°C. Before NMR analysis, urine was briefly thawed at room temperature and centrifuged at 5,900 ***g*** for 2 min. 15 µl of urine was mixed with 15 µl sterile water and 45 µl of 0.2 M phosphate buffer [pH 7.4, containing 20% D_2_O to provide an NMR field frequency lock and 0.5 mM trimethylsilyl-propionic acid (TSP) for internal chemical shift reference]. The sample was transferred to a 1.7 mm OD capillary tube, capped by a Teflon adapter and placed within a 5 mm micro NMR sample tube (New Era, USA). Urinary NMR *s*pectra were acquired using a JEOL 500 MHz Eclipse+ NMR spectrometer. Water presaturation was used for all data acquisitions. The following data acquisition parameters were used: spectral width 15 ppm, pulse angle 90°, acquisition time 4.36 s, relaxation delay 2 s, 32K data points and 256 data collects. The resulting free induction decay was zero filled and multiplied by an exponential function corresponding to 0.3 Hz line broadening prior to Fourier transformation. The NMR spectra were manually phased using the JEOL Delta software before being exported to the BioRad KnowItAll^®^ v9.0 (Bio-Rad, USA) software package for principal components analysis (PCA).

### NMR data analysis

Prior to statistical analysis all NMR spectra were baseline corrected to a 4th degree polynomial, zero filled by a factor of 2 and referenced with the TSP peak set to 0.00 ppm using KIA version 9.0 (Bio-Rad). The resonances attributable to residual water and urea (δ 4.60-6.40 ppm) were excluded from further analysis.

### NMR peak identification

The identification of NMR resonances to kynurenate and xanthurenic acid were confirmed by comparison of NMR data sets with solutions of kynurenic acid (10 mM in water) and xanthurenic acid (10 mM in methanol-d_4_). Further confirmation was obtained by adding 17 μM of kynurenic acid and 0.3 mM xanthurenic acid solutions to a specific urine sample to confirm peak overlap with the relevant NMR resonances.

### Statistical analysis

Data collection, summary calculations and descriptive statistics were carried out using Microsoft Excel 2010. To determine position and dispersion of data, descriptive statistics (mean±s.e.m.) were first applied. All data within this paper are presented as mean±s.e.m. unless otherwise stated. Statistical analysis was carried out using software GraphPad Prism *v*6.

Several measures were taken repeatedly over time from the same individual mouse. These include body weight and plasma hormone concentrations. Application of two-way ANOVA with repeated measures with post-hoc Bonferroni correction was applied to account for factors of time, genotype and individual variation within animals. Results were considered significant at *P*<0.05.

Effects of genotype on clinical chemistry parameters, hematology data and islet histology were determined by one-way ANOVA followed by post hoc Bonferroni correction. Differences were considered significant at critical *P<*0.0166, as is suitable for three comparisons (i.e. between *Afmid^+/+^*, *Afmid^tm1b/+^* and *Afmid^tm1b/tm1b^* groups) or in instances where only *Afmid^+/+^* and *Afmid^tm1b/tm1b^* data were available then effects were determined by Student's 2-tailed *t*-test. Results were considered significant at *P*<0.05.
